# Genome-wide association of trajectories of systolic blood pressure change

**DOI:** 10.1186/s12919-016-0050-9

**Published:** 2016-10-18

**Authors:** Anne E. Justice, Annie Green Howard, Geetha Chittoor, Lindsay Fernandez-Rhodes, Misa Graff, V. Saroja Voruganti, Guoqing Diao, Shelly-Ann M. Love, Nora Franceschini, Jeffrey R. O’Connell, Christy L. Avery, Kristin L. Young, Kari E. North

**Affiliations:** 1 Department of Epidemiology, University of North Carolina, Chapel Hill, NC 27514 USA; 2 Department of Biostatistics, University of North Carolina, Chapel Hill, NC 27514 USA; 3 Department of Nutrition, and UNC Nutrition Research Institute, University of North Carolina, Kannapolis, NC 28081 USA; 4 Department of Statistics, George Mason University, Fairfax, VA 22030 USA; 5 School of Medicine, University of Maryland, Baltimore, MD 21201 USA

## Abstract

**Background:**

There is great interindividual variation in systolic blood pressure (SBP) as a result of the influences of several factors, including sex, ancestry, smoking status, medication use, and, especially, age. The majority of genetic studies have examined SBP measured cross-sectionally; however, SBP changes over time, and not necessarily in a linear fashion. Therefore, this study conducted a genome-wide association (GWA) study of SBP change trajectories using data available through the Genetic Analysis Workshop 19 (GAW19) of 959 individuals from 20 extended Mexican American families from the San Antonio Family Studies with up to 4 measures of SBP. We performed structural equation modeling (SEM) while taking into account potential genetic effects to identify how, if at all, to include covariates in estimating the SBP change trajectories using a mixture model based latent class growth modeling (LCGM) approach for use in the GWA analyses.

**Results:**

The semiparametric LCGM approach identified 5 trajectory classes that captured SBP changes across age. Each LCGM identified trajectory group was ranked based on the average number of cumulative years as hypertensive. Using a pairwise comparison of these classes the heritability estimates range from 12 to 94 % (SE = 17 to 40 %).

**Conclusion:**

These identified trajectories are significantly heritable, and we identified a total of 8 promising loci that influence one’s trajectory in SBP change across age. Our results demonstrate the potential utility of capitalizing on extant genetic data and longitudinal SBP assessments available through GAW19 to explore novel analytical methods with promising results.

## Background

There is great interindividual variation in systolic blood pressure (SBP) as a result of the influences of several factors, including sex, ancestry, smoking status, medication use, stress, socioeconomic status, and, especially, age [1]. Studies estimate strong genetic effects when examining SBP cross-sectionally (h^2^ = 0.42), with even higher estimates for longitudinal measures of SBP change (h^2^ = 0.57) [2]. Yet, there are few published large-scale genetic studies that have leveraged longitudinal measures of blood pressure (BP) [3, 4]. Newer studies that have partitioned heterogeneous phenotypes into meaningful subphenotypes have proven useful for the identification of novel genetic susceptibilities and have allowed for previously missing heritability to be detected in complex disease traits (eg, cancer, autism, schizophrenia) [5–8]. These studies suggest that methodological innovations that identify homogenous subphenotypes may be useful for characterizing longitudinal BP change, yet this avenue remains largely unexplored in the genetic literature.

One possibility for identifying subphenotypes of BP change is through a group-based trajectory analysis. Trajectories can take advantage of longitudinal data to create a new outcome variable to summarize a unique component of the change in the phenotype, thereby minimizing trait heterogeneity, and allowing for a new trait to be considered in quantitative genetic analyses, which may allow for the identification of clinically relevant genetic biomarkers for disease progression and prognosis, such as hypertension [9]. Structural equation modeling (SEM) can help to show if certain covariates relate to the change trajectories, which will decrease bias while increasing precision and accuracy in genome-wide association (GWA) study analyses. Therefore, this study aims to conduct a GWA study of SBP change trajectories using data available through the Genetic Analysis Workshop 19 (GAW19). We first used previously identified BP single nucleotide polymorphisms (SNPs) to perform SEM and model the assumed underlying relationships between variables while taking into account potential genetic effects. Then these SEM results were used to inform a semiparametric latent class growth modeling (LCGM), which was used to identify distinct groups of SBP change trajectories within the population, using a widely available statistical package (PROC TRAJ for SAS) [10, 11], for use in the GWA study analyses.

## Methods

### Materials

GAW19 data were provided by the Type 2 Diabetes Genetic Exploration by Next-generation sequencing in Ethnic Samples (T2D-GENES) Consortium Project 2. Participants’ genetic and phenotypic data were drawn from 959 individuals from 20 Mexican American families as part of the San Antonio Family Studies (SAFS) [12].

### Phenotypes

Our analysis focused on SBP, where SBP was corrected for BP-lowering medication by adding a constant to all SBP measures that reported medication use (SBP + 15 mm Hg) [13]. Only individuals with a minimum of 2 SBP measures who did not exhibit greater than 3 standard deviations of change in any SBP measure were used in the final trajectory and association analyses (*N* = 683). Each time-varying measure was collected at 4 time points across 17 years; however, all subjects with at least 2 measurements were included as these methods assume missing data are missing completely at random.

### Single nucleotide polymorphism selection

Genome-wide data for 472,049 SNPs genotyped at Texas Biomedical Research Institute on the Illumina Infinium Bead chips: HumanHap550v3, supplemented with HumanExon510Sv1; Human660W-Quadv1; Human1Mv1; and Human1M-Duov3 arrays on odd-numbered autosomes were provided for analysis. All SNPs used in the analyses were filtered for Mendelian errors, monomorphic SNPs [12], and Hardy-Weinberg equilibrium. Merlin was used to impute missing genotypes. For the SEM model prediction, we extracted previously identified SBP, diastolic blood pressure (DBP), and pulse pressure–associated SNPs [14–19] from previous GWA studies data and considered each SNP separately. SNPs were only included in SEM if each genotype had at least 30 individuals. The full genetic panel, including imputed genotypes, was used in the trajectory association analyses.

### Structural equation modeling

**Fig. 1:**
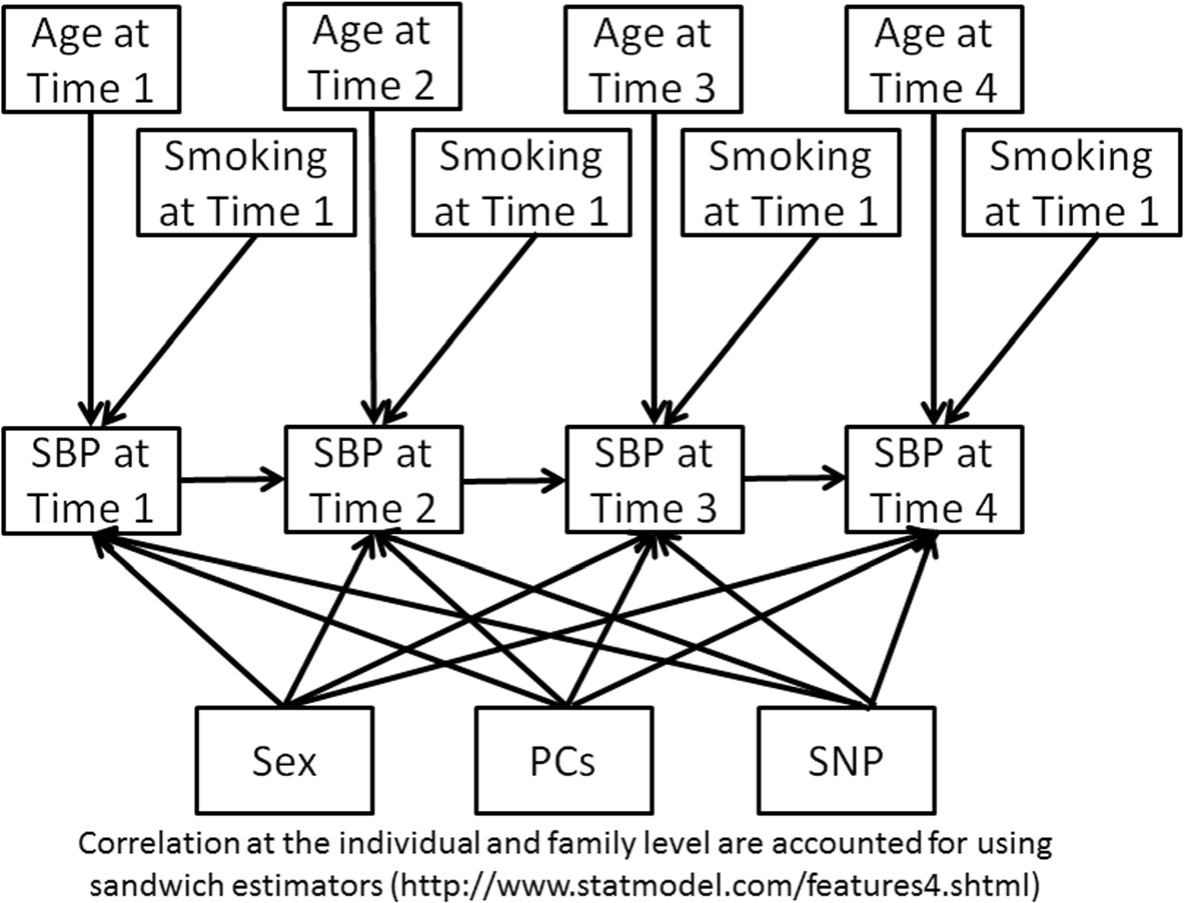
Full SEM model. Diagram illustrating the full SEM model and all direct and indirect pathways included PCs, principal components

We used SEM to identify covariates to include in estimating the SBP change trajectories. SEM is used here to determine if potential covariates are associated with trajectory class membership or associated with deviations from the assigned class-specific trajectory to better determine how and if covariates should be used in the LCGM. For the SEM component (Mplus v7.11), we defined a structure a priori to evaluate at what time points certain covariates directly and indirectly impacted SBP in separate SNP models, using previously identified SNPs extracted from the full genome-wide data. A lag effect for SBP was included to allow for current values to be related to values of the time point immediately prior, thereby allowing for covariates to have an indirect effect on SBP through their impact on a previous time point. Figure 1 illustrates the full SEM model, including direct and indirect covariate effects tested. Generalized estimating equations were used within the SEM framework to account for the correlation within an individual and within a pedigree. Root mean square error of approximation (RMSEA) as well as comparative fit index (CFI), and Tucker-Lewis index (TLI) were examined to ensure appropriate model fit. Good model fit was defined as RMSEA <0.06, and CFI and TLI values close to 1.0 [20–22]. Only those variables that were significant in at least 2 different SNP models (Fig. 1) and at multiple time points for each of these models were included in the final LCGM analysis; however, other variables were considered for adjustment in the association analysis (eg, principal components [PCs] to control for ancestry).

### Latent class growth modeling

We used a semiparametric LCGM approach to identify distinct groups of developmental trajectories [10, 23, 24]. Identification of SBP trajectories was done assuming a censored normal distribution using multivariate mixture model implemented in PROC TRAJ in SAS version 9.2. This model assumes that given class membership, the repeated measurements for the *i* ^*th*^ individual are independent [10]. SBP was modeled across age, including data from all 4 time points. Significant covariates in the SEM models were included to account for their impact on an individual’s trajectory. Final models were selected using the Bayesian information criterion (BIC) as well as practical considerations such as group size (*N* > 25) [25], uniqueness, and interpretability of classes. During model selection all trajectories were assumed to follow the same order polynomial. However, once the best model was selected with regards to the number of classes and overall order of polynomial, we assessed the impact of the order of the polynomial for each class on model fit and retained only significant intercept, linear, and quadratic terms. The identified trajectory classes were assigned at the individual level, based on the class with the highest predicted posterior probability of class membership [25].

### Genome-wide association analyses

Each LCGM identified trajectory group was ranked based on health risk, defined as the average number of cumulative years as hypertensive (ie, members of group 1 exhibiting the fewest number of hypertensive years and group 5 the greatest). We used PC scores to model differences in ancestral contributions among study participants. PCs were calculated using the unrelated founders and a subset of 28,156 independent (*r* ^*2*^ < 0.2) SNPs [26]. The resulting top 4 PCs were included as additional covariates in association analyses, as previous analyses found these sufficient for controlling population substructure [27]. We used SOLAR (Sequential Oligogenic Linkage Analysis Routines) [28] to estimate heritability. For GWA analysis on the trajectory group membership variables, we used MMAP, an open source software package written in C for genetic association analysis in both population-based and family data using linear mixed models. MMAP uses variance components within the mixed model framework to account for relatedness between individuals. For GWA analyses, we assumed an additive genetic model, and included the first 4 PCs as fixed effects to control for population structure [29]. We conducted both pairwise GWA analyses between groups with group 1 as the referent group. Also, to take advantage of the full data set, we conducted a GWA treating the rank ordered trajectories as a continuous trait in MMAP, coded as 1 through 5. Results were deemed genome-wide significant (GWS) when *p* < 1.3E−7 and suggestive when *p* < 1.6E-6, based on previous San Antonio Family Studies [30] simulations.

## Results

### Structural equation modeling

The final SEM models included a total of 19 previously identified BP-associated SNPs available in the GAW19 data set to identify mediators and effect modifiers to include in the identification of trajectory classes. Four of the established BP SNPs displayed a nominally significant (*p* < 0.01) association with SBP at 1 or more time points, and were investigated further for potential effect mediators. The SEM led to the inclusion of sex in the LCGM and the consideration of PCs in the GWA (data not shown).

### Latent class growth modeling

**Fig. 2:**
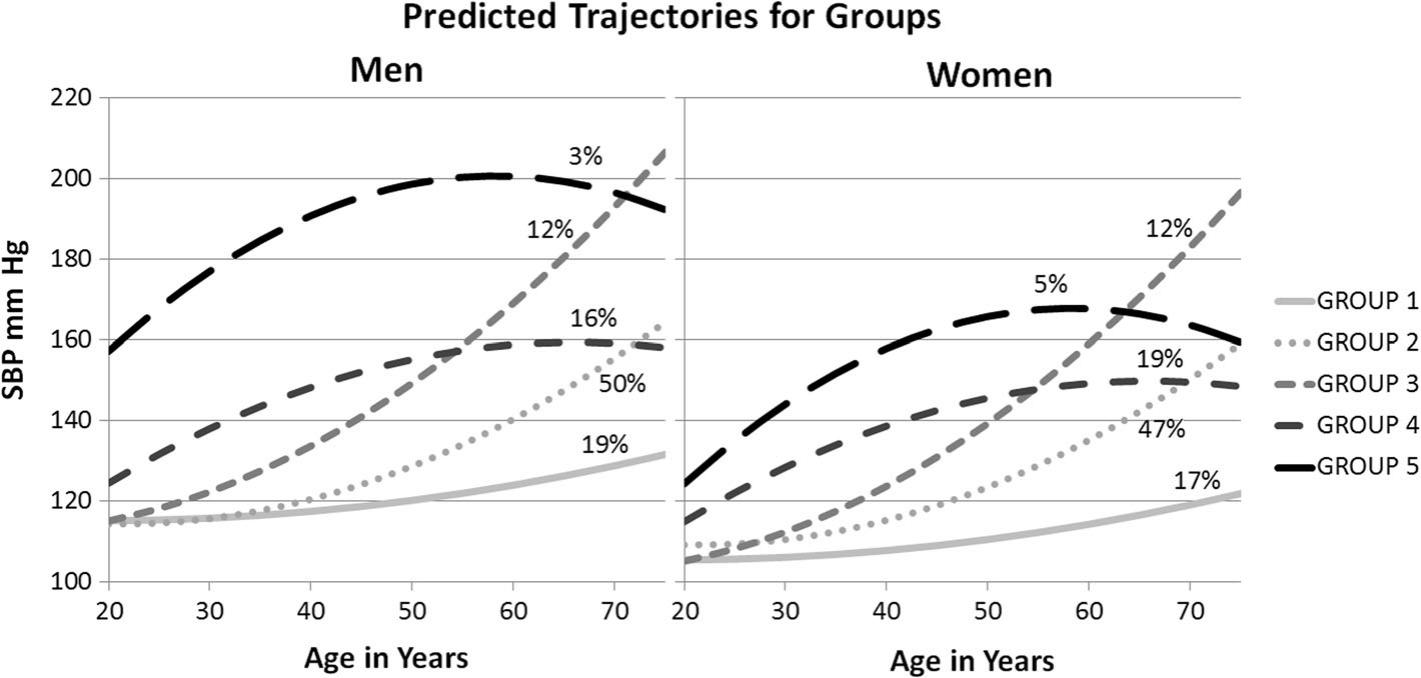
Fitted trajectories. Fitted trajectories by sex for each group identified in the LCGM analysis

As the difference in SBP trajectories between men and women were not of primary interest for this study and sex impacted SBP directly and indirectly at multiple time points in the SEM, we adjusted for sex at each time point in the LCGM. Class prediction was performed requesting 1 to 6 groups, with the highest BIC resulting from assignment of 5 groups with age as a quadratic function in all classes (Fig. 2).

### Heritability and covariate selection

Table 1 summarizes the results of the heritability estimates and displays the significant PCs for each pairwise group comparison. The heritability estimates range from 0.12 to 0.94, standard errors (SE) from 0.17 to 0.40 (Table 1). PC2 and PC3 were both significant predictors of group comparisons in 1or more models.

**Table 1:** Heritability and GWA study results

Marker	CHR:POS	Nearest Gene	E/O Allele	EAF	Trajectory Class 2 (N up to 416)			Trajectory Class 3 (N up to 224)			Trajectory Class 4 (N up to 187)			Trajectory Class 5 (N up to 136)			Ordinal (N up to 633)		
					h^2^ = 0.12 [0.17] *P* = 0.2117			h^2^ = 0.57 [0.28] *P* = 0.0137			h^2^ = 0.94 [0.34] *P* = 0.0044			h^2^ = 0.87 [0.40] *P* = 0.0256			h^2^ = 0.23 [0.09] *P* = 0.0007		
					B	SE	P	β	SE	P	β	SE	P	β	SE	P	β	SE	P
rs17112252	5:151103540	*ATOX1*	A/C	0.96	0.47	0.08	***1.0E-08***	0.28	0.10	7.2E-03	−0.02	0.10	8.8E-01	0.08	0.10	4.2E-01	0.032	0.170	8.53E-01
rs17133935	7:44704204	*OGDH*	G/A	0.18	0.06	0.04	1.5E-01	0.05	0.07	5.0E-01	0.12	0.07	1.0E-01	0.30	0.06	**3.2E-07**	0.308	0.081	1.62E-04
rs7857537	9:100337976	*GABBR2*	C/T	0.03	0.27	0.11	1.2E-02	0.46	0.21	2.8E-02	0.50	0.19	7.6E-03	0.89	0.17	**5.1E-07**	0.571	0.188	2.50E-03
rs4756864	11:16762663	*PLEKHA7* ^a^	C/T	0.42	0.14	0.03	8.2E-06	0.15	0.05	3.0E-03	0.21	0.06	2.1E-04	0.26	0.05	**1.7E-07**	0.262	0.063	3.28E-05
rs2159537	17:12316500	*MYOCD*	G/A	0.12	−0.03	0.06	6.0E-01	0.20	0.08	9.0E-03	0.10	0.08	2.6E-01	0.35	0.07	4.2E-06	0.472	0.097	**1.44E-06**
rs630539	17:37946870	*NAGLU*	C/T	0.02	0.28	0.13	2.5E-02	0.51	0.25	4.4E-02	0.55	0.28	4.9E-02	0.89	0.17	**1.0E-06**	0.650	0.225	3.93E-03
rs11203213	21:43034914	*PDE9A*	T/C	0.42	0.02	0.03	5.4E-01	0.16	0.05	1.0E-03	0.09	0.06	1.1E-01	0.17	0.05	4.6E-04	0.262	0.063	3.62E-05
rs762407	21:44005874	*PDXK*	A/G	0.42	0.06	0.03	8.3E-02	0.17	0.05	9.1E-04	0.25	0.05	**1.0E-06**	0.11	0.05	3.3E-02	0.319	0.064	**7.03E-07**

Results of the heritability estimates for the pairwise comparison of group membership with group 1 as the referent group and the ordinal analysis are presented for each test, as h^2^ [SE], *p* value. Results of the GWA study analyses including all SNPs that reached genome-wide significance (*p* < 1.3E-7) (highlighted in bold-italic) and those SNPs that reached suggestive significance (*p* < 1.6E-6) (highlighted in bold)

*CHR:POS* chromosome and base pair position, *E/O* effect/other, *EAF* effect allele frequency estimated from total analytical sample, *P p* value, *SE* standard error of beta estimate

^a^Previously identified SBP-associated locus [11, 19]

### Genome-wide association

**Fig. 3:**
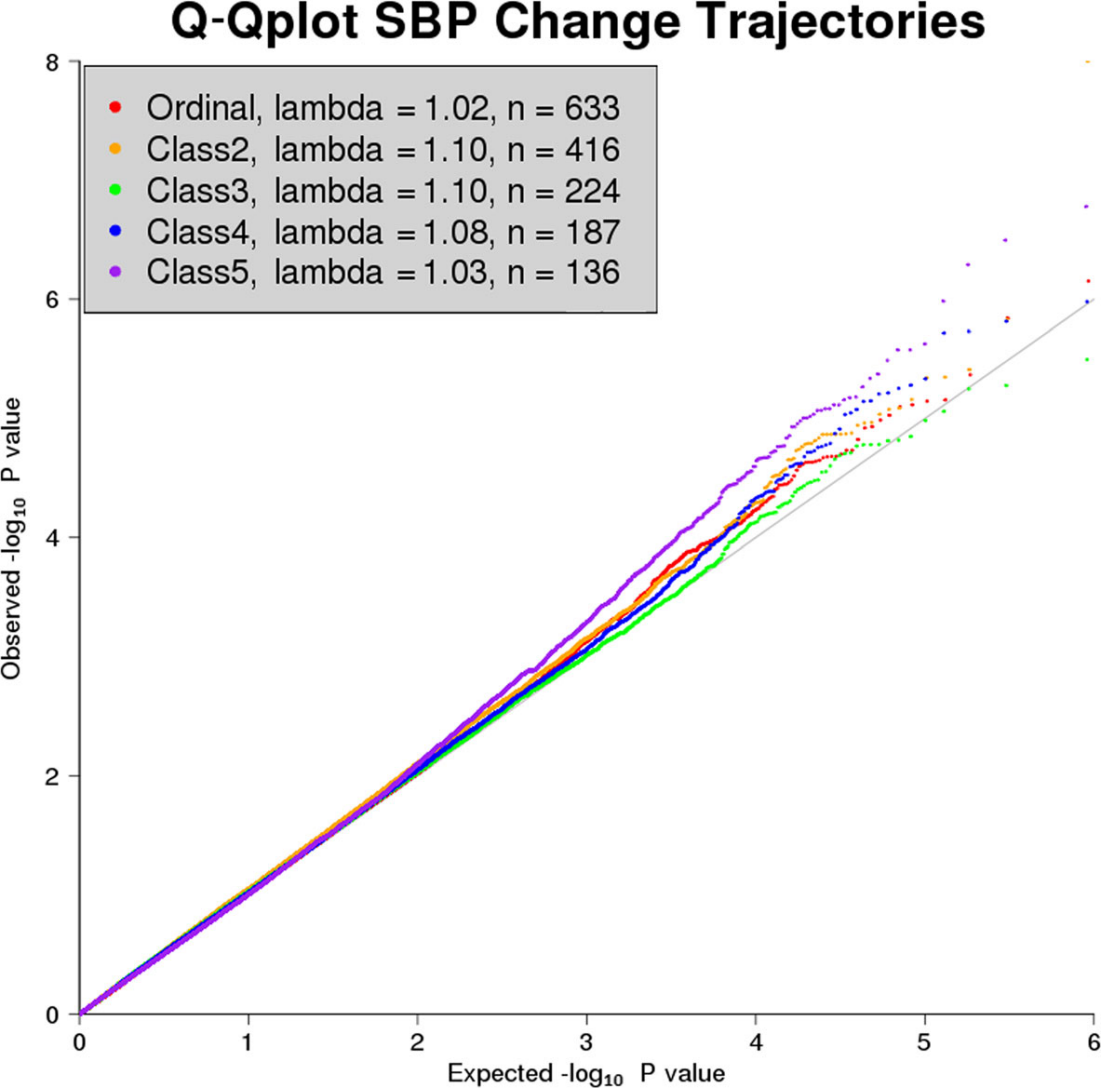
Q-Q Plot. Quantile-quantile (Q-Q) plot of SNP associations, color-coded by association test, and including sample size (N) and lambda values

A quantile–quantile (Q-Q) plot of the final GWA results for all 5 approaches exhibits no strong evidence for genomic inflation (Fig. 3). After filtering on minor allele frequency (MAF) of greater than 1 %, association analyses identified one GWS (*p* < 1.3E-7) SNP near the *ATOX1* gene (rs17112252, *p* = 1E-8) for the pairwise association analysis of trajectory group 2 (see Table 1). Although no other GWA study analysis resulted in a GWS association, an additional 7 SNPs reached suggestive significance (*p* < 1.6E-6) in 1 or more GWA studies, including rs4756864 for the group near the *PLEKHA7* gene, only 96 kb from rs381815, a known SBP-associated SNP identified in Europeans, although in low linkage disequilibrium (*r* ^*2*^ = 0.04, CEU [Utah residents with ancestry from northern and western Europe] HapMap r22) [15].

## Discussion

We used the SEM model to identify particular time points where certain covariates were more or less informative and direct and indirect effects, which may modify genetic influence on longitudinal SBP in Mexican Americans. While age, sex, and smoking status are known to influence cross-sectional SBP in non-genetic analyses, we found that only age and sex had significant effects on SBP change while accounting for genetic effects of known BP loci. We identified 5 unique trajectories using longitudinal data to create a new outcome variable for genetic association testing. These identified trajectories are significantly heritable, and we identified a total of 8 promising loci that influence one’s trajectory in SBP change across age.

One limitation of using the SEM methods to inform covariate selection is that we used previously identified GWA SNPs associated with SBP, DBP, and pulse pressure from cross-sectional data and identified in European, Asian and African descent populations, which may not generalize to Mexican Americans. These loci may not fully account for possible genetic effects in our longitudinal analysis of Mexican Americans and miss possible modifiers to longitudinal genetic effects. The assumption of scale in the ordinal analysis is also a limitation, as health risk may not be equal between the ranked groups. Lastly, another limitation is that GWA study analyses were only performed on odd-numbered chromosomes. We expect that additional loci associated with SBP change will be identified in other regions of the genome in future studies.

## Conclusions

The majority of investigations into the genetic underpinnings of SBP do not take advantage of the wealth of longitudinal data available in many large cohort studies. To address this important research gap, we have capitalized on longitudinal assessments of SBP and extant genetic data available through GAW19 to evaluate a novel and unique statistical data analysis. This study assesses how genetic variants, environment, and behavior effect progression of SBP and will provide new data to examine the pathogenesis of hypertension. The innovative methods considered herein have been used to identify several promising variants associated with SBP change trajectories and can be easily implemented in GWA for a wide range of longitudinally assessed traits.
